# Complete mitochondrial genome of Schizothorax davidi (Teleostei: Cypriniformes: Cyprinidae)

**DOI:** 10.1080/23802359.2019.1668729

**Published:** 2019-09-24

**Authors:** Shuo Feng, Zhifang An, Yue Wang, Jian Liang

**Affiliations:** State Key Laboratory of Plateau Ecology and Agriculture, Qinghai University, Xining, China

**Keywords:** *Schizothorax davidi*, mitochondrial genome, phylogenetic analysis

## Abstract

This study provides a mitochondrial complete genome of *Schizothorax davidi*. The complete genome is 16,576 bp in length with an A + T content of 54.9%, which contains 13 protein-coding genes, 22 tRNA genes, and 2 rRNA genes. The phylogenetic analysis indicated that *S. davidi* is closely related to *Schizothorax lissolabiatus*. These results contribute to explore the adaptation strategy of *S. davidi* on the conditions of Qinghai-Tibet Plateau.

*Schizothorax davidi* belongs to the genus *Schizothorax*, within the subfamily *Schizothoracinae* (Cypriniformes, Cyprinidae), which was mainly distributed in the Jialing River and Minjiang River of the upstream of the Yangtze River (Ding 1994). China is the country with the largest distribution of *Schizothoracinae* fishes in the world (Wu and Wu 1991), and the germplasm resources have become a research hotspot. In the last few years, some research of the phenotypic, physiological and molecular level (Qi et al. 2008; Zhao et al. 2008), but there was lack of the genomic information of the *Schizothoracinae* fishes. Therefore, establishing a complete mitochondrial genome sequence will contribute to explore the adaptation strategy of *S. davidi* on the conditions of Qinghai-Tibet Plateau.

In this study, *S. davidi* were collected from the Ruoergai County (Gansu, China; 102°37′E, 34°05′N) and voucher specimens (ZK-Ruoergai-001) were deposited at the Museum of State Key Laboratory of Plateau Ecology and Agriculture, Qinghai University. The sample was frozen in liquid nitrogen immediately after collection and stored at −80 °C. The complete mitogenome sequence of *S. davidi* was deposited into GenBank database with accession number (MK861919). The whole length of the mitogenome of *S. davidi* is 16,576 bp, with an A + T content of 54.9%. The whole mitogenome contains 13 protein-coding genes, 22 tRNA genes, and 2 rRNA genes.

We constructed a phylogenetic tree (Figure 1) by RAxML 8.1.17 (Stamatakis 2014) to represent the phylogenetic relationships between *S. davidi* and other Schizothorax species. The results demonstrate that *S. davidi* had the highest homology with *Schizothorax lissolabiatus*, followed by *Schizothorax lantsangensis* (Figure 1).

**Figure 1.**
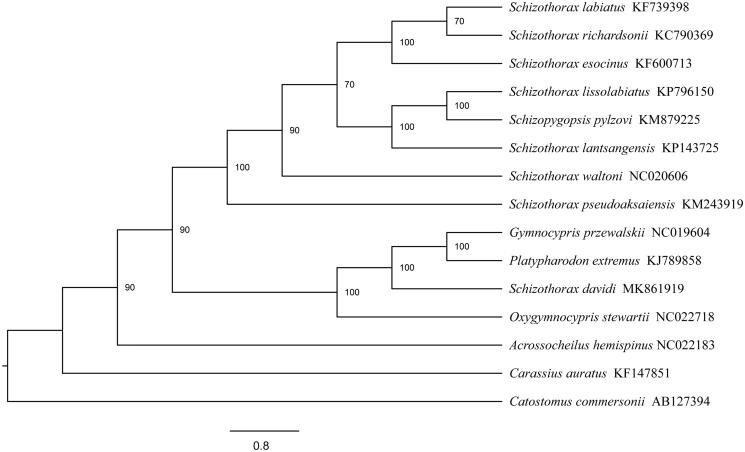
The maximum likelihood tree of the *Schizothorax davidi* and 14 other species with GenBank accession numbers.
